# Evaluating hospital websites in Kuwait to improve consumer engagement and access to health information: a cross-sectional analytical study

**DOI:** 10.1186/s12911-018-0660-4

**Published:** 2018-09-24

**Authors:** Dari Alhuwail, Zainab AlMeraj, Fatima Boujarwah

**Affiliations:** 1 Department of Information Science, College of Computing Sciences and Engineering, Kuwait University, Adailiya, Kuwait; 2 Health Informatics Unit, Dasman Diabetes Institute, Sharq, Kuwait

**Keywords:** Website evaluation, Accessibility, Usability, Social media, Consumer health informatics, Patient participation, Patient education

## Abstract

**Background:**

Current advances in information and communication technology have made accessing and obtaining health-related information easier than ever before. Today, many hospital websites use a patient-centric approach to promote engagement and encourage learning for better health-related decision making. However, little is known about the current state of hospital websites in the State of Kuwait. This study aims to evaluate hospital websites in Kuwait and offer recommendations to improve patient engagement and access to health information.

**Methods:**

This study employs a cross-sectional analytical approach to evaluate hospital websites in Kuwait in 2017. The websites of hospitals that provide in-patient services were identified through a structured search. Only active websites that were available in either English or Arabic were considered. The evaluation of the websites involved a combination of automated and expert- based evaluation methods and was performed across four dimensions: Accessibility, Usability, Presence, and Content.

**Results:**

Nine hospitals met the inclusion criteria. Most of the websites fell short in all four dimensions. None of the websites passed the accessibility guidelines. The usability of websites varied between hospitals. Overall, the majority of hospitals in Kuwait have rudimentary online presence and their websites require careful reassessment with respect to design, content, and user experience. The websites focus primarily on promoting services provided by the hospital rather than engaging and communicating with patients or providing evidence-based information.

**Conclusions:**

Healthcare organization and website developers should follow best-practices to improve their websites taking into consideration the quality, readability, objectivity, coverage and currency of the information as well as the design of their websites. Hospitals should leverage social media to gain outreach and better engagement with consumers. The websites should be offered in additional languages commonly spoken by people living in Kuwait. Efforts should be made to ensure that health information on hospital websites are evidence-based and checked by healthcare professionals.

## Background

In today’s connected world, consumers are increasingly using the Internet to seek health-related information [1–4]. This increase is catalyzed by the surge of mobile technologies and affordable access to the World Wide Web [5, 6], thus creating opportunities for healthcare organizations to engage their consumers via informative and educational online platforms. Researchers argue that patients, and their potential role in managing their conditions, have been the least utilized resource in healthcare [7, 8]. Evidence suggests that patients who are more actively involved in their own healthcare experience better outcomes and do not burden the healthcare system with high costs [9]. This is far more feasible as health Information Technology (IT) solutions can facilitate better patient-centered care via improving healthcare processes, clinical outcomes, responsiveness to patients’ needs and preferences, shared decision-making, communication between patients and clinicians, and access to medical information [10, 11]. Therefore, many healthcare organizations today are leveraging health IT tools and solutions, such as websites, to better engage, involve, and educate patients [12, 13].

Typically, hospital websites are a good reference for general information about a hospital, its services, and its clinicians [14]. These websites could also serve as a good medium to educate and inform patients, their families, and the general public about diseases, procedures, medications, and healthy lifestyles [15–17]. However, despite the promise of greater information availability, patient-focused healthcare websites have not advanced as quickly as compared to other industries [18]. This is especially true for the Gulf Cooperation Council (GCC)^1^ region. Hospital websites in Kuwait, Saudi Arabia and the United Arab Emirates for example, offer limited access to necessary education and support resources for patients’ wellbeing and are not tailored to the customs, culture, and language of those living in the region [19–21]. In recent years, Kuwait has had significant movements towards electronic government across its agencies [22]. Yet, little is known about the levels of participation of healthcare facilities, including hospitals, in these initiatives. After all, hospitals are an essential part of successful government interaction with the citizens through the web.

Therefore, it becomes important to understand the current state of hospital websites and evaluate them to improve access to health information as well as patient engagement. This study aims to thoroughly evaluate hospital websites and offer recommendations to improve patient engagement and access to health information. We examine hospital websites in the State of Kuwait as a sample from the GCC region and offer recommendations for healthcare organization and website developers to improve the quality of information, authority and objectivity, coverage and currency, as well as the design of their websites. The countries of the GCC have very similar healthcare systems, face similar challenges, and share a history of cooperation between them [23, 24]. As such, any lessons learned about the status of hospital websites in Kuwait will in turn benefit the entire region.

### Healthcare system in Kuwait

Through its constitution, the State of Kuwait is obligated to provide free universal coverage to its citizens while expatriates pay nominal fees for non-emergency health services and government-subsidized medications. In Kuwait, approximately 80% of healthcare services are rendered by the public sector through the Ministry of Health, which acts as the owner, operator, regulator, and financer across the country. The government provides these services through 92 primary care centers, 6 general hospitals, and 13 specialized hospitals and centers [25]. While Kuwait has low rates of infectious and communicable diseases, the non-communicable and chronic diseases account for 73% of total deaths [26]. In 2015, Kuwait’s public health expenditure was more than 7% of the total government expenditure [27]. It is clear from the expenditure data that the public healthcare system in Kuwait is heavily reliant on treatment as opposed to preventative services [26]. The alarming rates of non-communicable and chronic diseases pose serious challenges for the government [28] and demand better patient engagement and education via accessible means in todays’ connected world (i.e. online health-related websites). Hence this research evaluates the status of hospital websites in Kuwait across multiple dimensions for their consumer engagement and access to health information and services.

## Methods

### Design and approach

This study employs a cross-sectional analysis approach [29] to evaluate hospital websites in the State of Kuwait. Initially, the list of hospital websites in Kuwait was compiled from the Ranking Web of World Hospitals [30]. An additional manual search was conducted in “Google Search” to locate additional websites of hospitals not listed by the Ranking Web of World Hospitals. For websites to be considered for inclusion, the website must be active, reachable, available in either the Arabic or English language, and associated with a hospital recognized by the Ministry of Health in Kuwait which offers multi-day in-patient admissions and services.

### Evaluation

The researchers followed analogous procedures employed by similar studies in analyzing the hospital websites [18, 31–33]. Data collection about the websites began in April 2017 and took approximately 1 week to complete. Data collection was conducted within the week to ensure that information collected across the websites and their pages were not affected by their availability or major modifications. The researchers evaluated English (and Arabic if available) pages of the included websites.

Prior to collecting the data and evaluating the websites, the researchers determined appropriate checklists to evaluate the following dimensions: Accessibility, Usability, Presence, and Content. Similar checklists that were used by other researchers were used as a foundation for the checklists used in this study. The developed checklists were carefully crafted to ensure compatibility with the nature of the healthcare system in Kuwait, the cultural and social norms. For example, the researchers took into account the popular social media platforms used in Kuwait and included them as part of the checklist for Presence.

As shown in Table 1, these checklists involve a set of criteria that could either be checked automatically or by an expert user [18, 31, 32]. The expert-based website evaluations were conducted by two-experts according to the checklists, their scores were compared and in the event that there was disagreement, these differences were discussed and reconciled between them.

**Table 1:** Evaluation methods adopted for the study

Dimension	Criteria	Evaluation mode
Accessibility		
	AChecker [35]	Automated
Usability		
	LIDA [36]	Expert-based
Presence		
	Modified Checklist [18]	Expert-based
Content		
	HON [38]	Expert-based
	Readability [39]	Automated

#### Accessibility

This dimension evaluates the website’s accessibility according to criteria set by the World Wide Web Consortium (W3C). The Content Accessibility Guidelines (WCAG) [34] at Level AA were chosen for this study. To evaluate the accessibility of the hospital websites, an automated tool named AChecker was used [35]. Three commonly visited web pages were chosen per hospital and evaluated: The home/landing page, the clinicians’ directory, and contact page. The resulting dataset included 26 web pages (9∗3–1) with one page found to be under construction. For each web page, the automatic tool tests the HTML source code for adherence to the WCAG 2.0 guidelines based on standard principles to ensure the content is: Perceivable, Operable, Understandable, and Robust. The AChecker tool divides observed problems into three categories:

- (K) Known problems that are identified as obscuring accessibility.
- (L) Likely problems that are identified as probably obscuring accessibility.
- and (P) Potential problems that could not be identified by the tool and requires the help of an expert to determine their nature.

#### Usability

This dimension is an assessment of the website’s ability to present information to its consumers in a useful way. It focuses on the clarity of the information presented, how consistent the website is overall, and whether it offers good functionality or not. The Minervation LIDA Instrument V1.2 [36], an instrument developed specifically to assess healthcare websites, was used to evaluate the websites. The researchers assessed the websites’ (a) clarity and appropriateness of language used, (b) consistency of website design and ease of navigation, and (c) functionality of the site in providing users with the right tools to find what they need without overburdening them with unnecessary functions.

#### Presence

This dimension is an assessment of the website’s digital presence and online reach-ability through different channels and mediums. Researchers developed a modified checklist based on prior studies [18]. This checklist considered the presence of the hospital in the most accessed social media channels by people living in Kuwait [37]. The researchers collected information about the number of Facebook page likes, number of Twitter and Instagram followers, the number of YouTube channel subscribers. In instances where the website did not specifically provide a link to its social media accounts, the researchers performed the search manually and directly through the social media websites.

#### Content

This dimension is an assessment of the website’s overall content quality without taking into consideration the technical limitations. The websites’ content is assessed using (i) the Health On the Net (HON) Foundation’s Site Evaluation checklist [38], which is an overall assessment of the reliability of health-related information available on the Internet, and (ii) the readability scores using the Fletch-Kincaid Reading Ease and Grade Level scales [39].

## Results

In total, 15 websites were identified by the researchers. After applying the inclusion/exclusion criteria, four websites were excluded because they were inactive at the time of the data collection and two additional websites were for hospitals that did not offer in-patient admissions. Only nine hospitals with unique website domains were included in the study. This included six private and three government hospitals. Refer to Table 2 for detailed demographic information about the evaluated hospitals [40].

**Table 2:** Hospital demographic information

Hospital ID and type	Outpatient visits	No. of beds	Age (years)	No. of employees
H1 - Private^a^	15,439	117	54	733
H2 - Private^a^	12,255	106	50	529
H3 - Private^a^	17,335	185	9	860
H4 - Private^a^	7481	61	11	267
H5 - Private^a^	6282	105	8	478
H6 - Private^a^	6433	64	19	344
H7 - Government	7723	189	64	650
H8 - Government	20,219	375	52	965
H9 - Government	3525	769	68	716

^a^Full adoption of electronic health records

### Accessibility

The results of the evaluation indicate that there was no single website that passed the WCAG 2.0 [Level AA] accessibility guidelines.

Table 3 shows the number of identified problems averaged across the set of three pages per hospital according to the four accessibility principles. Notable is the proportion of perceivable errors, which are higher in comparison to others. The tool also identified a total of 3034 Known errors, 35 Likely problems, and 9483 Potential problems across all pages as presented in Table 4. Interestingly, the identified ‘Known’ problems have the largest impact and are relatively easy to resolve. Descriptions of these problems are summarized in Table 5.

**Table 3:** The average known hospital website problems per principle

ID	Perceivable	Operable	Understandable	Robust
H1	317	7	14	1
H2	74	3	2	0
H3	32	32	8	1
H4	612	120	6	1
H5	15	1	0	0
H6	0	0	2	0
H7	6	4	2	0
H8	9	1	2	0
H9	59	0	2	0

**Table 4:** AChecker results^a^

ID	Landing page				Find a clinician				Contact us			
	Result	K	L	P	Result	K	L	P	Result	K	L	P
H1	F	320	10	640	F	316	0	639	F	382	0	506
H2	F	46	0	440	F	149	0	1052	F	42	0	227
H3	F	124	0	1434	F	19	0	240	F	30	0	336
H4	F	143	10	425	F	556	0	682	F	94	1	439
H5	F	21	0	383	F	9	0	282	F	17	0	304
H6	F	2	0	10	F	2	0	20	F	2	0	10
H7	F	30	6	115	F	3	0	44	F	3	0	38
H8	F	14	3	80	F	13	0	88	F	10	0	108
H9	F	8	1	103	F	174	0	148	NA	NA	NA	NA

^a^*F* Fail, *P* Pass, *K* Known, *L* Likely, *P* Potential problems

**Table 5:** Top 15 known accessibility issues

List of common *known* problems (Level AA)	Count
Element “img” missing “alt” attribute	1776
Element “i” or italic used	142
On-mouseover event handler missing on-focus event handler	103
Image used as anchor is missing valid “alt” text	80
Insufficient contrast between text color and its background	69
Script not keyboard accessible – on-mouse-out missing on-blur	61
Anchor contains no text	53
Label text is empty	47
Input element type of “text” has no/missing associated label	38
Document language not identified	33
Document has invalid language code	32
Input element type of “text” has no text in label	21
Header nesting error	19
Element selected missing an associated label	18

#### Perceivable

Overall, the majority of the errors found fall under the principle Perceivable. The most common perceptual errors across all tested hospital pages as shown in Table 5 are “image elements missing alternate attributes” (1776 errors), “multiple i (italic) elements used” (142 errors), and “lack of contrast between text and background colors” (69 errors). Additionally, images, plug-ins and embedded media all require alternative text such as captions, sign language, and audio descriptions, with a clear indication of the language used. There are 80 errors pertaining to this problem alone.

#### Operable

Being able to navigate and find content is very important. Developers are advised to make all functionality, and features added to the websites, available from the keyboard. For example, “mouse over missing event handler” (103 errors) and “scripts not accessible by the keyboard” (61 errors) should be removed to allow users enough time to read and use content. Missing navigation methods to help find content and location on the websites such as “missing titles and anchor texts” (over 100 errors) can be easily overcome.

#### Understandable and robust

A total of 166 errors were found in relation to understandability. For a website to be readable and understandable, assistive technologies must recognize the document language and the language code. Every hospital website evaluated has at least one missing document language and language code identification (33, 32 errors consecutively), as well as multiple missing labels and label texts (101 errors) that often lead to confusion for webpage visitors.

### Usability

**Fig. 1:**
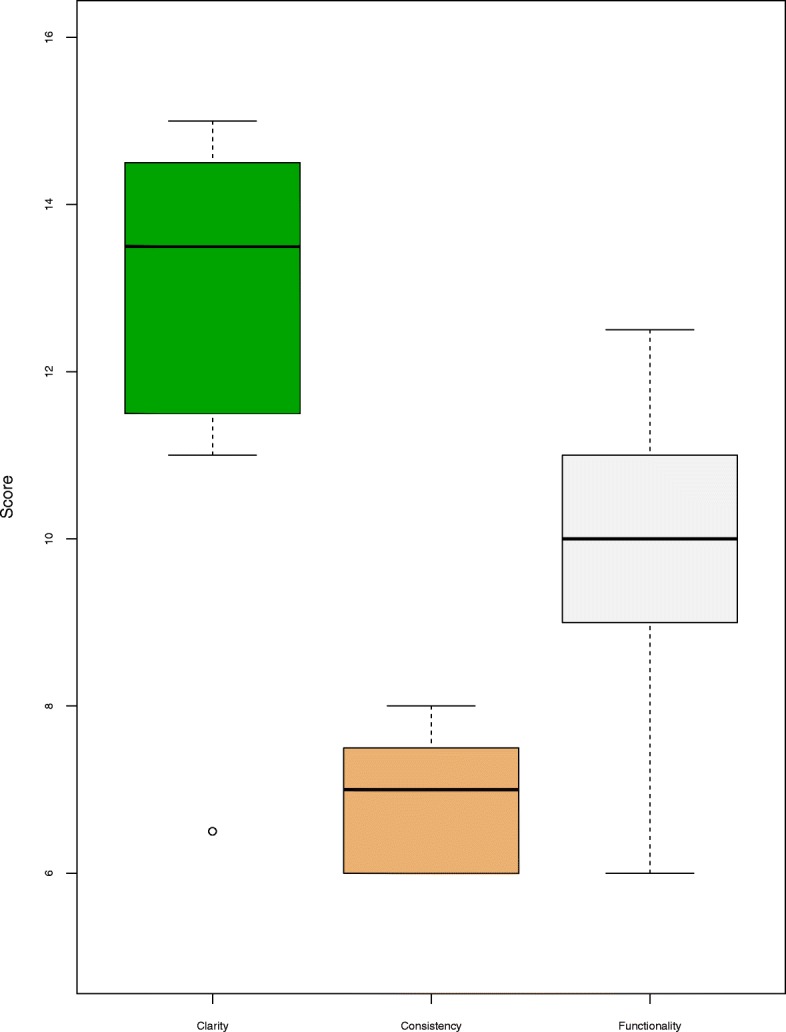
Boxplot of hospital website usability scores

The lowest scoring website was that of hospital (H6). This hospital has a fully flash-based website. This made the site completely unusable without a plug-in and reduced the usability scores across all 3 sections of the checklist. The highest scoring hospital overall (H1), did not receive the highest score in all 3 sections, however, it did perform relatively well in all 3 sections. Refer to Fig. 1 for overall usability evaluation scores and Table 6 for the specific scores per website.

**Table 6:** Hospital website usability scores^a^

ID	Clarity (18)	Consistency (9)	Functionality (15)	Overall (42)
H1	13.5	8	12.5	34
H2	14.5	6.5	11	32
H3	15	7	10.5	32.5
H4	14	8	12.5	34.5
H5	11.5	6	9	26.5
H6	13.5	6	10	29.5
H7	11	7.5	9	27.5
H8	14.5	7.5	10	32
H9	6.5	6	6	18.5
*Min*	*6.5*	*6*	*6*	*18.5*
*Max*	*15*	*8*	*12.5*	*34.5*
*Median*	*13.5*	*7*	*10*	*32*

^a^Total score for each usability sub-category is reported in parenthesis

Within the Clarity sub-dimension, on average the websites had higher scores for appropriateness of the language, and lower scores for ease of navigation. All websites, except H6, achieved a score of 6 or higher in the Consistency sub-dimension. In the Functionality subdimension, only 3 websites provided an effective search facility. On the other hand, the highest scoring criteria across the sites was the availability of effective browsing facilities.

### Presence

The majority of websites had presence on social media platforms, namely Facebook, Twitter, YouTube, and Instagram (Refer to Table 7 for more detailed information). It is worth nothing that government hospital websites did not leverage social media to expand their online presence. Only one government hospital had Twitter and Instagram accounts with less than 700 followers to each account.

**Table 7:** Hospitals’ presence across social media networks

ID	Facebook	Twitter	YouTube	Instagram
H1	38,872	6971	652	21,700
H2	8743	924	25	21,800
H3	1698	*N/A*	40	*N/A*
H4	9111	14,200	3046	24,500
H5	2374	144,000	21	59,500
H6	217	497	0	14,200
H7	*N/A*	659	10	571
H8	*N/A*	*N/A*	*N/A*	*N/A*
H9	*N/A*	*N/A*	*N/A*	*N/A*

### Content

Most of the evaluated hospitals (6) had their websites available in both Arabic and English. Overall, the English versions of the websites appeared more comprehensive and contained more information.

#### Authoritative information

Consistently across all the evaluated websites, some of the health and medical information was not attributed to an author. Only two websites provided information that was not authoritative in nature. Also, there were no clear statement that particular sections of the website contained information from non-medically qualified individuals or organizations*.*

#### Complementarity of information

None of the websites declared that the information provided on their sites was designed to support and not replace the relationship that exists between the site visitor and his or her existing healthcare provider.

#### Statement on privacy

Only one website declared a clear privacy and confidentiality policy regarding the use and storage of e-mail addresses, personal, and medical information via the website. It was not clear whether any of the websites respected the legal requirements, including those concerning medical and personal information privacy, that apply in Kuwait.

#### Medical information and its sources

While the majority of the websites (7) provided medical information for patients and site visitors in the form of original content, none of the websites provided a modification date, both for the website as a whole or for the pages that contained medical information. Specifically, two of these websites provided medical information from outside source (e.g. information about Diabetes and pregnancy) without properly citing the source. One website offered electronic versions of its printed hospital magazine, which was in the Arabic language. Another website offered bilingual (Arabic/English) patient leaflets organized by medical specialties that were available at the hospital. Another website offered only English health education information. Two additional sites embedded health education videos (one website in Arabic and one website in English) from their channels on YouTube.

#### Justifiability of the information

Eight websites contained original content and did not make any claims relating to the benefit or performance of a specific medical treatment, commercial product or service. Only two websites made such claims based on personal research or opinions. *Disclosures:* All the websites included in this study did not clearly describe any potential conflicts of interest including funding sources and its advertising policy.

#### Content readability

The websites’ median score for the Flesch-Kincaid Reading Ease test was 41.4. and at the ninth-grade level, exceeding the recommended sixth-grade level. The Flesch-Kincaid readability tests indicates how difficult a passage in English is to understand by specifying the grade level the text is recommended for. As the reading ease score increases, the recommended grade level is lowered. Refer to Table 8 for more detailed scores per website.

**Table 8:** Websites’ Flesch-Kincaid readability scores^a^

ID	Reading ease	Grade level
H1	44.2	9
H2	38.2	9
H3	62.7	5.4
H4	35.2	9.1
H5	−6.9	14.9
H6	78.9	4
H7	46.4	8.7
H8	63.4	5.5
H9	33.4	10.1
*Median*	*41.42*	*9.65*

^a^Scores are reported for only for pages written in English. For reading ease scores, 100–90 = very easy, 30-zeor = very difficult

## Discussion

To the best of our knowledge, no prior study evaluated Kuwaiti hospital websites thoroughly by examining the accessibility, presence, content, and usability dimensions. Overall, the results suggest that the majority of hospitals in Kuwait require careful evaluation of their websites’ design and content. Interestingly, while governmental hospitals in Kuwait are older in age and provide care services to a large number of patients, they are behind in adopting and maintaining well-designed websites. This could be attributed to their overall slow adoption of health IT solutions, for example electronic health records, as illustrated in Table 2.

It is interesting to note that despite local residents’ extensive reliance on major well-established government hospitals for healthcare, the websites and online presence of these institutions are rudimentary. Our findings also highlight that the evaluated websites focus primarily on promoting services provided rather than engaging and communicating with patients or providing evidence-based health information. With the growing demand from consumers to locate health information online [41], it becomes essential for hospitals in Kuwait to create and maintain well-designed and engaging websites that adhere to international and national standards [18]. In the remainder of this section we discuss implications for practice and based on our finding offer recommendations relevant to hospital administrators and website developers.

### Designing for accessibility

As evident from the results, due to the low level of conformance to the W3C WCAG 2.0 accessibility guidelines, it is necessary for Kuwaiti hospitals to consider the issues highlighted in this evaluation. Failing to adhere to these kinds of standards have recently been considered a form of discrimination against persons with disabilities [42–44]. To improve website accessibility, web developers should:

- Experiment with different representations of text whether it be visual, audio, tactile representations or a combination of the three. For example, a blind person can understand a picture if the browser reads out an attached alternative text and analogously a deaf person can understand a picture or audio file if there is a visual alternative on the page.
- Provide users the ability to control the contrast between foreground text and background color on the webpages to help users with poor vision read the text on the page.
- Avoid the use of bold and italic text as assistive technologies fail to identify these styles. As an alternative, substituting styles with fonts that are easier to read are recommended.
- Validate pages and close open tags to help assistive technologies perform parsing will improve robustness of the websites and compatibility across Internet browsers.

Considering the types of errors discussed in the results, it is feasible to consider that the hospital website developers lack awareness of Web accessibility standards and tools such as WGAC 2.0. Therefore, it is important to increase web developers’ awareness and knowledge of established accessibility standards and guidelines through appropriate training. Future studies could investigate Web developers’ awareness of existing accessibility standards and practices in more detail.

### Empowering usability

As demonstrated by the website usability scores (refer to Table 6), it is interesting to note that the government hospitals scored among the lowest compared to the private hospitals. Our results are consistent with similar studies [45] suggesting the need to improve usability. It is recommended that hospitals conduct usability sessions with patients regularly to enhance usability. Additionally, there is a great opportunity for government hospitals to use their websites to help educate patients as well as provide electronic services such as booking appointments or contacting medical professionals. It is possible that improving website usability could reduce demand for services and help hospitals better manage wait times for services. With well-designed hospital websites, healthcare providers can engage patients and guide them to quality, evidence-based health information [3].

### Curating health information

As evident from the findings, across all hospital websites, many webpages that contained health and medical information were not attributed to an author. Therefore, it is important that any medical or health advice provided online should be given by medically-trained and qualified professional only [46]. Clinicians should play a more active role by asking patients what they learned from online resources and where they obtained the information from, including hospital websites [47]. Clinicians can then better assist patients in a shared and collaborative decision-making process that paints a complete picture of for example the risks and benefits of treatment options [48].

All hospital websites can benefit from carefully and thoroughly reviewing the content of their websites and ensuring that it is evidence-based and conforms to the HON principles. The evidence reveals that many hospital webpages that may have contained advertising or promotional health information were indistinguishable from clinical information. As the Internet becomes crowded with biased health and medical information, hospital websites need to clearly label advertisements and promotional information about procedures clearly as to not cloud the judgement of patients [16]. Hospitals need to apply more scrutiny and stricter advertising regulations to eliminate the imbalance between clinical information and the promotional information which can negatively impact the patient decision-making. Additionally, there is a need for authoritative and regulatory bodies (e.g. the Ministry of Health and Public Authority for Food and Nutrition) to take a more active role in certifying health information on hospital websites.

### Reaching diverse populations

Despite the fact that nearly 70% of Kuwait’s population are expatriates who may not speak Arabic or English [49], the evaluated websites were available in only the Arabic and/or English languages. The hospital websites should be offered in additional languages commonly spoken by people living in Kuwait, such as Hindi, Urdu, and Tagalog [21]. Additionally, the results reveal that the evaluated websites are written at readability levels above the recommended reading levels for the multiethnic, multicultural general public in Kuwait [50]. The website and its content’s readability are a concern since many of Kuwait’s population are nonnative English (or Arabic) speakers. While the American Medical Association and the National Institutes of Health recommend that the readability of patient education materials should not exceed a sixth-grade reading level [51, 52], no specific reading level recommendation is available for the GCC context [53]. Therefore, hospitals should carefully develop the content on their websites in a manner that is easy to read, understand and comprehend by the general population.

Similar to other findings [54], the results demonstrate the modest presence of hospital websites, especially government hospitals, on social media platforms. Given that most of healthcare services are provisioned by the public sector, it is essential that Kuwaiti government hospitals leverage social media to gain better outreach and engagement with patients. Doing so will also help hospitals increase their market share and improve patient experience and engagement [55, 56].

The results clearly showcase that private hospitals in Kuwait are doing better with regards to having a more professional and engaging website. Perhaps this is due to the fierce nature of competing with other hospitals over funds and to attract more patients with private insurance or those that can pay out-of-pocket. Whereas the government hospitals generally do not compete with any other hospitals, neither private or government.

### Comparison with prior work

This study followed similar evaluation approaches to earlier studies conducted in different parts of the world as illustrated in Table 9. The evidence from our study has many similar findings with the evidence from the listed studies and it points out that many hospital websites need careful evaluation and rework to improve the access and quality of information presented on the website as well as improve the website visitors’ engagement and the services provided online. Globally, it also appears that many hospitals still have low presence on social media and are not fully leveraging and embracing its power to engage patients. Distinctively, this study focuses on both public and private hospital websites in a specific geographic region, whereas some of the other studies focused on specific diseases. While the Internet and the World Wide Web has no boundaries, the contextual determinants, i.e. the structure of the healthcare systems, culture, and customs, can be different among geographic regions and locations.

**Table 9:** Summary of similar Studies^a^

Study	Country or region	Website type	Sample size	Year	Major findings
Maifredi et al. [57]	Italy	Italian hospitals	763	2009	High percentage of hospitals do not provide an official website. Very few websites provide information to increase credibility of hospital and user confidence in institution.
Liu et al. [58]	China	Public hospitals	23	2009	Most websites show good performance in content, a normal performance in function and design, but bad performance in website management & usage.
Selig et al. [59]	Germany	Burn centers	44	2010	Websites offer a good overview about institution’s online services via numerous multimedia-based elements. However, the quality of specific information for burn patients is relatively poor.
Orlowski et al. [60]	USA	Heart failure websites	5	2011	Websites written at high readability levels (8-9th grade), but easily navigated.
Weber et al. [21]	GCC	General health websites	925	2012	Evaluating HON standards, approximately less than 10% of websites post privacy policy or authorship of information. Over 50% of websites provide a date for information. Only 1.7% report advertising policy and 23.5% disclose sponsorships.
Huerta et al. [32]	USA	Hospital websites	2407	2013	Management of hospitals’ online presence is not adequate.
Huerta et al. [18]	USA	Children hospital websites	153	2014	Wide range of websites’ score with no perfect website suggesting room for meaningful improvements.
Raj et al. [61]	India	General health websites	32	2014	Most websites have average quality, especially in usability. Many websites written at high readability levels.
Salarvand et al. [33]	Iran	Public hospitals	59	2016	Overall, low level quality of the websites evaluated.

^a^Some studies were focused on general health information and were not specific to hospitals

### Study strengths and limitations

Similar to other research, this study has several strengths and limitations. The included nine hospitals in this study represent approximately 40% (*N* = 22) of the healthcare institutions in Kuwait that provide healthcare services beyond primary care. Carefully studying other healthcare organizations, including primary care centers, out-patient clinics, and physician offices will help provide more insights about the overall online presence of healthcare organizations in Kuwait. The researchers performed additional web searches of hospital websites to be included in the study and did not rely solely on the original listing of hospital websites in Kuwait by Ranking Web of World Hospitals. Additionally, while patient perspectives were not in the scope of this study, the researchers, who are informatics experts, recalled their experiences as patients when navigating the websites. Future studies should solicit feedback directly from patients and consumers seeking information from hospital websites. Lastly, the results can be informative for hospitals in Kuwait when evaluating how their current and future websites will support patients’ informational needs. However, careful consideration of the specific context is required before directly assuming applicability of the results to all hospitals in the GCC or other Arab countries.

## Conclusions

The proliferation of the Internet as a source for health information presents a great opportunity for hospitals to better engage with their patients and improve their care experience. In this study, we provide a comprehensive assessment of nine Kuwaiti in-patient hospitals using automated and expert-based tools and evaluation methods. To the best of our knowledge, no prior study evaluated Kuwaiti hospital websites thoroughly by examining the accessibility, presence, content, and usability dimensions. Most of the websites fell short in all four dimensions. Overall, the majority of hospitals in Kuwait require careful reassessment with respect to design, content, and user experience. The websites focus primarily on promoting services provided by the hospital rather than engaging and communicating with patients or providing evidence-based information. Hospital administrators, public relations managers, and web developers can use the recommendations resulting from this study to improve their hospitals’ websites. Future studies can investigate the perceptions and opinions of patients and consumers in the broader GCC areas in terms of accessibility, usability, presence, and content of hospital websites.

## Abbreviations

GCC: Gulf Cooperation Council
HON: Health on the Net

## Footnotes

1 GCC countries include Saudi Arabia, Kuwait, the United Arab Emirates, Qatar, Bahrain, and Oman.
